# Development of an efficient protein expression system in the thermophilic fungus *Myceliophthora thermophila*

**DOI:** 10.1186/s12934-023-02245-5

**Published:** 2023-11-16

**Authors:** Jinyang Li, Yidi Wang, Kun Yang, Xiaolu Wang, Yuan Wang, Honglian Zhang, Huoqing Huang, Xiaoyun Su, Bin Yao, Huiying Luo, Xing Qin

**Affiliations:** State Key Laboratory of Animal Nutrition and Feeding, Institute of Animal Sciences, Chinese Academy of Agricultural Sciences, Beijing, 10093 China

**Keywords:** *Myceliophthora thermophila*, Protein expression, Promoter, 5’UTR, Signal peptide

## Abstract

**Background:**

Thermophilic fungus *Myceliophthora thermophila* has been widely used in industrial applications due to its ability to produce various enzymes. However, the lack of an efficient protein expression system has limited its biotechnological applications.

**Results:**

In this study, using a laccase gene reporting system, we developed an efficient protein expression system in *M. thermophila* through the selection of strong constitutive promoters, 5’UTRs and signal peptides. The expression of the laccase was confirmed by enzyme activity assays. The results showed that the *Mtpdc* promoter (P*pdc*) was able to drive high-level expression of the target protein in *M. thermophila*. Manipulation of the 5’UTR also has significant effects on protein expression and secretion. The best 5’UTR (NCA-7d) was identified. The transformant containing the laccase gene under the *Mtpdc* promoter, NCA-7d 5’UTR and its own signal peptide with the highest laccase activity (1708 U/L) was obtained. In addition, the expression system was stable and could be used for the production of various proteins, including homologous proteins like MtCbh-1, MtGh5-1, MtLPMO9B, and MtEpl1, as well as a glucoamylase from *Trichoderma reesei*.

**Conclusions:**

An efficient protein expression system was established in *M. thermophila* for the production of various proteins. This study provides a valuable tool for protein production in *M. thermophila* and expands its potential for biotechnological applications.

**Supplementary Information:**

The online version contains supplementary material available at 10.1186/s12934-023-02245-5.

## Background

The development of efficient, high-yield and low-cost protein production platforms has received increasing attention in recent years. Due to their excellent protein secretion capacity, filamentous fungi have been extensively studied and engineered for the production of a wide range of valuable proteins, including enzymes, pharmaceutical proteins, and industrial chemicals. The presence of a complex and efficient protein secretion system allows them to secrete large amounts of proteins into the extracellular environment [1]. This makes them ideal candidates for use as microbial cell factories for the production of high-value proteins. In addition, filamentous fungi are relatively easy to cultivate, have a fast growth rate, and can utilize a wide range of substrates, making them a cost-effective and sustainable option for industrial protein production [2].

The thermophilic filamentous fungus *Myceliophthora thermophila* is a highly proficient cellulose decomposer and one of the excellent producers of thermostable enzymes for industrial applications [3]. The genome sequence of *M. thermophila* ATCC 42464 was published in 2011 [4]. In recent years, various studies have been conducted with *M. thermophila*, such as biochemical characterization of enzymes [5–12], degradation of lignocellulosic biomass [4, 13–16], development of genetic tools [17–21], regulation of (hemi)cellulase gene expression [20, 22–28] and metabolic engineering for chemical production [29–36]. In addition, the industrial *M. thermophila* C1 has been developed into a protein production platform using strong inducible (cellobiohydrolase *cbh1*) or constitutive (chitinase *chi1*) promoters [37, 38]. This strain has been granted GRAS (Generally Recognized As Safe) status by the FDA (GRN No. 292), making it a suitable expression host for recombinant proteins used in the chemical, medical, food and other industries. Several other constitutive promoters have also been shown to drive gene expression in *M. thermophila*, such as the strong *Mttef1* (elongation factor 1-alpha) or *Mtpdc* (pyruvate decarboxylase) promoters [18, 26, 39–41]. However, their effects on protein secretion have not been fully investigated. Only the secretory expression of glucoamylases from *Myceliophthora heterothallica* (MhGlaA) and *Talaromyces emersonii* (TeGlaA) under the control of the *Mttef1* promoter (P*tef1*) has been reported [21, 40, 42].

Compared to other protein expression systems such as *Escherichia coli* and *Pichia pastoris*, *M. thermophila* is highly advantageous for protein expression due to its ability to degrade various plant biomasses through the production of (hemi)cellulases [16]. This feature makes it an attractive system for the direct production of desired proteins from plant biomass. In addition, its high-temperature fermentation capabilities at 45–50 °C can significantly reduce cooling costs and minimize susceptibility to contamination. Furthermore, *M. thermophila* has a unique glycosylation pattern that differs from *P. pastoris*, which confers greater specific activity and stability to the produced protein [43]. Moreover, the availability of genetic tools for *M. thermophila* enables efficient strain engineering for improved target protein production [21, 40, 42]. Collectively, these advantages make *M. thermophila* a promising candidate for protein expression, especially in applications that require efficient utilization of plant biomass and high-temperature fermentation.

The expression of recombinant proteins can be influenced by several factors, including the presence of expression elements such as promoters, 5’UTRs, and signal peptides [1, 44]. These elements play an important role in regulating gene expression and protein secretion in eukaryotic cells, but their effects on protein expression and secretion in *M. thermophila* have not been fully characterized. When a recombinant protein is produced in filamentous fungi, it’s laborious to screen positive transformants. Therefore, a simple and rapid detection method is needed. In this study, we used a gene reporting system based on the expression of the laccase gene to evaluate the effect of these elements on protein secretion. Despite the presence of laccase-encoding genes in its genome, *M. thermophila* shows no laccase activity under laboratory conditions. Transformants with laccase activity after overexpression of a laccase gene could be easily screened on plates using ABTS as a substrate, where a laccase-positive strain is indicated by a dark green halo or area around the fungal colony [37]. This simple and straightforward method has been effectively employed to determine the expression or its expression level of the laccase gene in *Gloeophyllum trabeum* [45], *Aspergillus niger*, *A. nidulans*, and *T. reesei* [46].

Among these elements, we found that the combination of the *Mtpdc* promoter (P*pdc*), the NCA-7d 5’UTR, and the native signal peptide (SP*lcc1*) resulted in the highest laccase activity. To demonstrate the versatility of this system, we used it to express several extracellular proteins, including a non-catalytic protein, MtElp1, from *M. thermophila* and four carbohydrate-active enzymes: cellobiohydrolase MtCbh-1, endoglucanase MtGh5-1, lytic polysaccharide monooxygenase MtLPMO9B from *M. thermophila* and glucoamylase TrGlaA from *T. reesei*. MtElp1 belongs to the cerato-platanin family, which is exclusively found in fungi and has been shown to enhances the hydrolysis of lignocellulosic materials [47]. All of these proteins were successfully overexpressed and secreted into the supernatant, indicating the potential of this system to produce a variety of proteins. Understanding the effects of these expression elements on protein production in *M. thermophila* could help to optimize the expression system and improve the yield and quality of recombinant proteins. This study provides valuable insights into the regulation of protein expression and secretion in *M. thermophila* and could pave the way for the development of more efficient and effective protein expression systems.

## Results and and discussion

### Efficient laccase production under glucose-repressed conditions

**Fig. 1:**
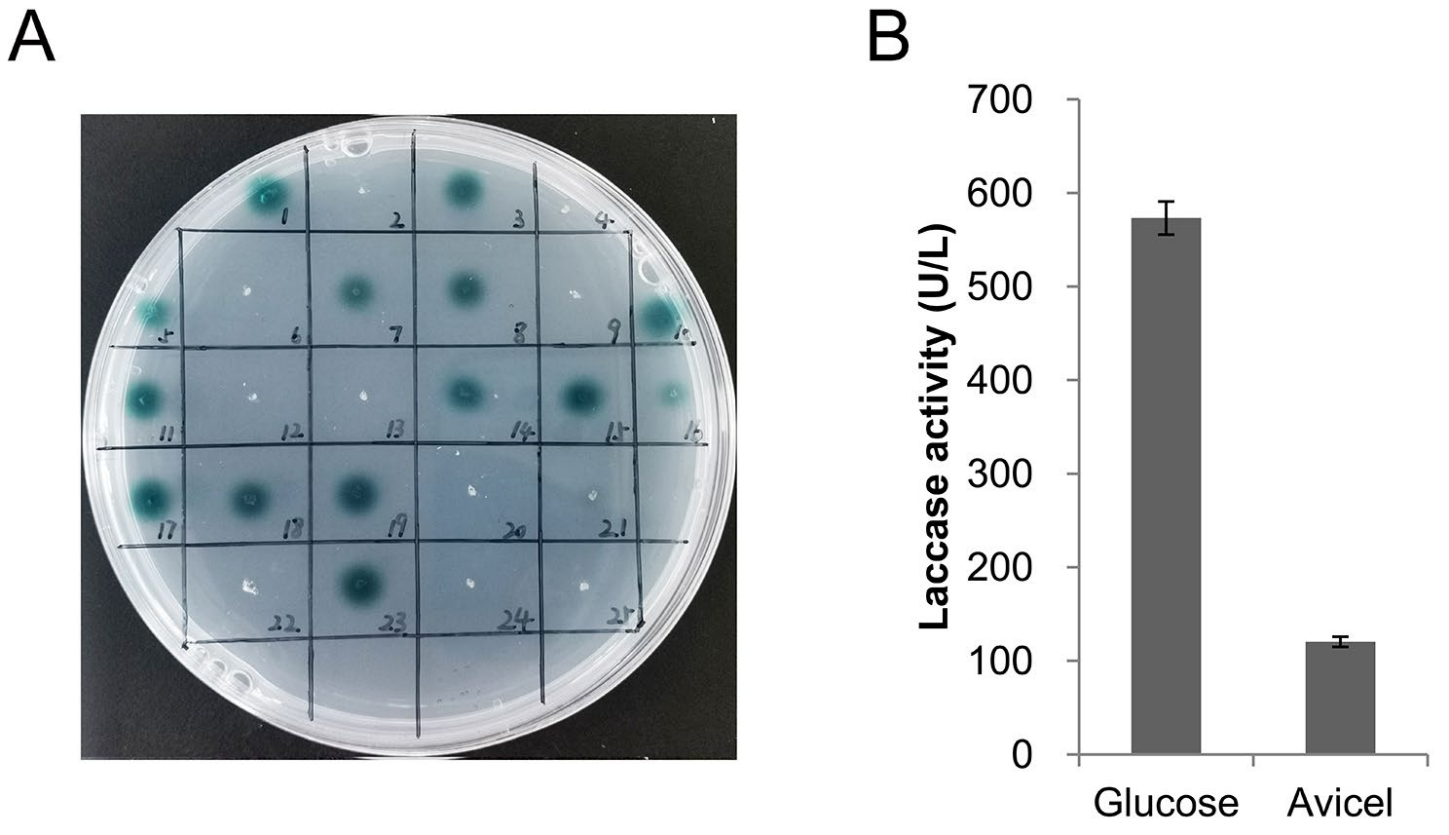
Efficient laccase production under glucose-repressed conditions. (**A**) *lcc1*-expressing strains were identified by the ABTS plate assay. Transformants were inoculated onto the MM medium supplemented with 1 mM ABTS and incubated at 37 °C for 3 h. (**B**) Laccase activity in culture supernatants of ∆*Mtalp1*::P*tef1*-*lcc1*-T*lcc1* strain after cultivation in fermentation medium containing 4% glucose or cellulase-inducing medium containing 2% Avicel (MM + 2% Avicel) at 45 °C and 200 rpm for 3 days

The laccase gene reporting system has been proven to be a cost-effective and rapid method for detecting extracellular laccase production [37, 45, 46]. Here, we used the laccase gene reporting system to screen for the expression elements that confer high levels of protein production in *M. thermophila*. First, the relative efficiency of different promoters in laccase production was determined. The expression cassette P*tef1*-*lcc1*-T*lcc1* was constructed for overexpression of *lcc1* under the control of a strong constitutive *Mttef1* promoter. To prevent potential proteolytic hydrolysis [46, 48], we utilized the ∆*Mtalp1* mutant as the host strain, as MtAlp1 is the primary extracellular protease [25]. Transformants expressing the laccase gene were identified by their ABTS-oxidizing activity (Fig. 1A). A transformant with an obvious blue halo was then inoculated into fermentation medium containing 4% glucose or cellulase-inducing medium (MM) containing 2% Avicel. After cultured for 3 days, the supernatant from the glucose medium exhibited a significantly higher laccase activity than that from the Avicel medium (Fig. 1B), demonstrating the efficient laccase production under glucose-repressed conditions. Therefore, the fermentation medium was used in the following experiment.

### Evaluation of constitutive promoter activities for laccase production

**Fig. 2:**
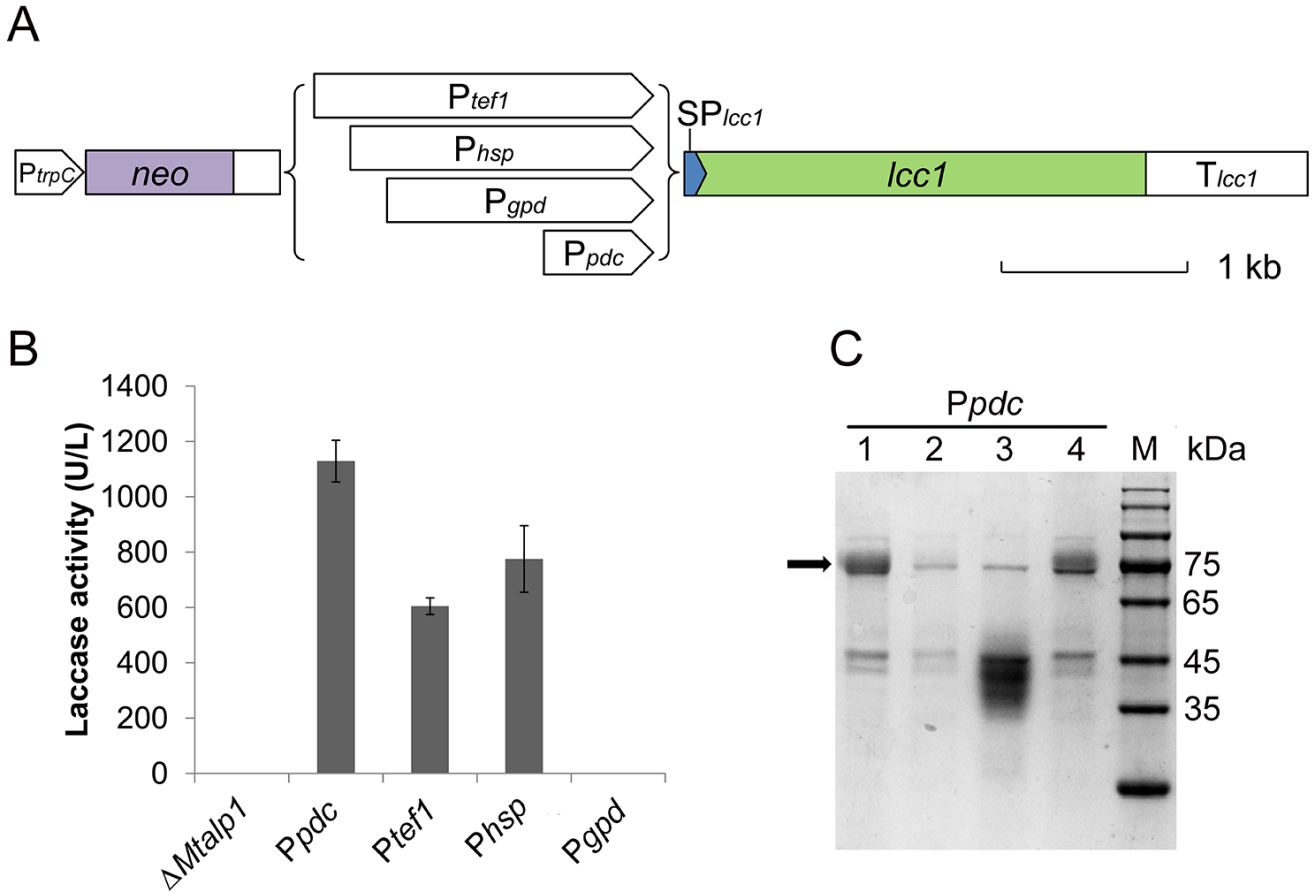
Expression of homologous laccase in *M. thermophila* under the control of different promoters. (**A**) Schematic representation of the gene expression cassettes for *lcc1*. (**B**) Laccase activity in culture supernatants of *M. thermophila* strains. (**C**) SDS-PAGE analysis of proteins secreted by transformants carrying (lane 1 and 4) or not carrying (lane 2 and 3) the P*pdc*-*lcc1* expression cassette. 50 µL of culture supernatant sample was loaded in each lane for Coomassie staining. The glycosylated MtL encoded by *lcc1* is indicated by an arrow. All strains were cultured in fermentation medium at 45 °C and 200 rpm for 3 days

Previous studies have demonstrated that the expression levels of *Mttef1*, *Mtgpd* (glyceraldehyde-3-phosphate dehydrogenase) and *Mtpdc* are significantly higher than other genes. In addition, we observed that the gene *Mthsp30*, which encodes a putative 30 kD heat shock protein, also exhibits a constitutively high expression pattern [23, 26, 31]. To assess the impact of these promoters on protein secretion, we constructed an expression cassette containing the laccase-encoding gene *lcc1* under the control of each promoter (Fig. 2A). After introduction into the ∆*Mtalp1* mutant, the transformants were assayed for laccase production. The results showed that the promoter has a significant effect on laccase production. When the *Mtgpd* promoter (P*gpd*) was used, none of the transformants showed laccase activity, which could be attributed to the removal of two introns in the 5’UTR of *Mtgpd*. And the P*pdc* promoter outperformed the other two promoters in terms of laccase expression and secretion, with approximately 1200 U/L of laccase activity being achieved (Fig. 2B). Interestingly, the *Mthsp30* promoter (P*hsp*) was more efficient than the commonly used P*tef1* promoter, suggesting that the *Mthsp30* promoter is a novel candidate for gene overexpression. The MtL was easily visualized on the SDS-PAGE gel and showed a single band of ~ 80 kDa (Fig. 2C), which is consistent with the previous study [49].

### Enhancing laccase production by manipulating the 5’UTR

**Fig. 3:**
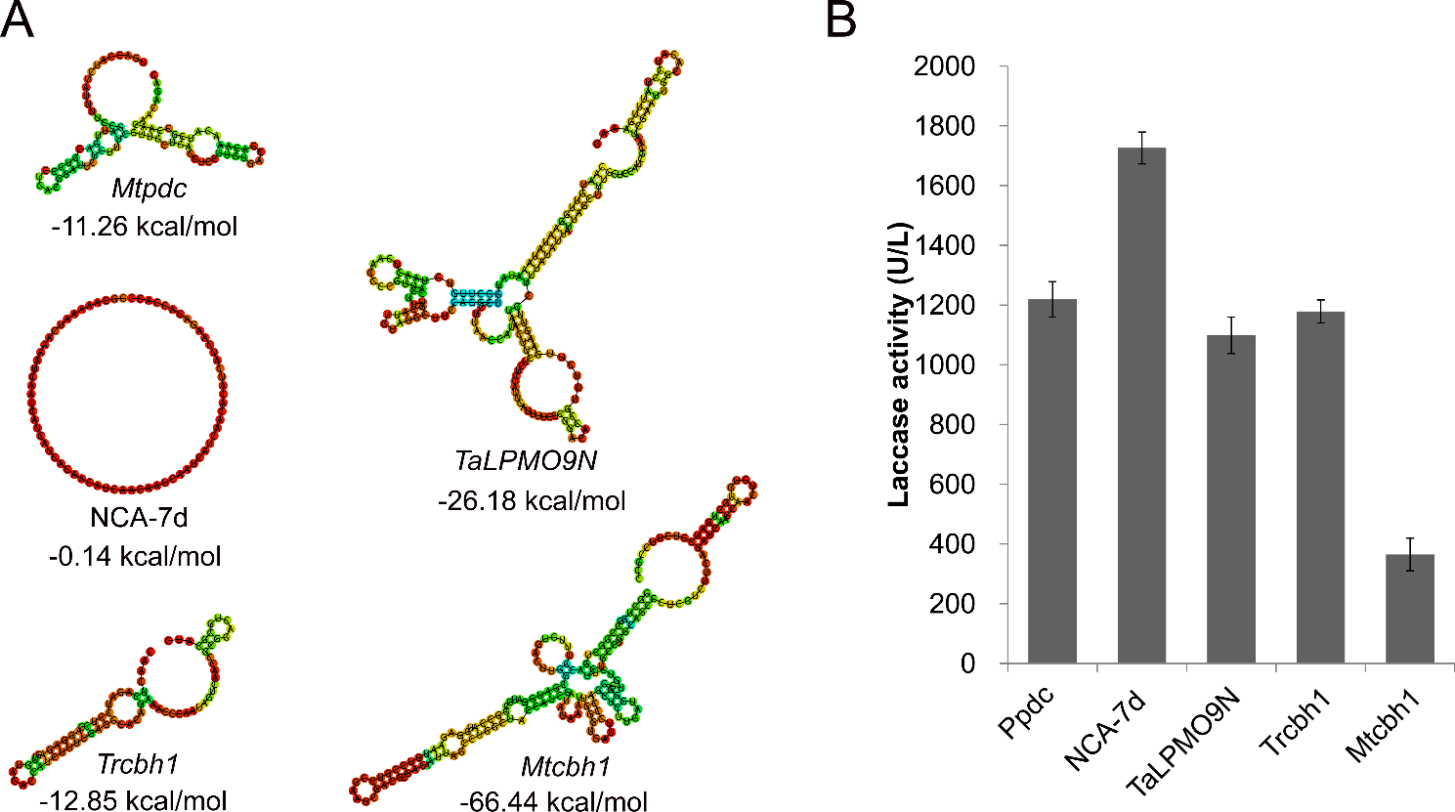
Influence of 5’UTR on laccase production. (**A**) Secondary structures prediction of the 5’UTR from different sources. Minimum free energy (MFE) secondary structures were predicted by RNAfold web server (http://rna.tbi.univie.ac.at). (**B**) Laccase activity in culture supernatants of *M. thermophila* strains after cultivation in fermentation medium at 45 °C and 200 rpm for 3 days

In order to express a protein effectively, an mRNA requires several essential components, including a 5’ cap, 5’ untranslated region (5’UTR), protein-encoding sequence, 3’ untranslated region (3’UTR), and the polyadenosine tail. Among these components, the 5’UTR is a unique regulator for protein translation [50]. In this study, we aimed to evaluate the efficiency of the 5’UTR in laccase production by selecting four 5’UTRs with different minimum free energy (MFE). These included a synthetic 5’UTR NCA-7d [51] and 5’UTRs of *Trcbh1*, *Mtcbh-1*, and *TaLPMO9N*, in addition to that of *Mtpdc* (Fig. 3A).

The results indicated that the use of NCA-7d 5’UTR led to a 41% increase in relative laccase activity, compared to *Mtpdc* 5’UTR (Fig. 3B). However, no significant difference was observed when *Trcbh1* 5’UTR or *TaLPMO9N* 5’UTR was used, while the lowest laccase activity was observed with *Mtcbh-1* 5’UTR (Fig. 3B). The laccase activity was found to be consistent with the minimum free energy (MFE) of the 5’UTR, except for *TaLPMO9N* 5’UTR. NCA-7d, with an MFE of **− 0**.14 kcal/mol, was found to be the most efficient in laccase production, while *Mtcbh-1* 5’UTR, with an MFE of **− 6**6.44 kcal/mol, was the least efficient. This finding is consistent with previous studies showing that NCA-7d is the optimal 5’UTR for luciferase expression in Hep3B and 293T cells [51], indicating its potential universality in gene expression. Therefore, NCA-7d was selected as the optimal 5’UTR for further experiments.

### Multiple signal peptides function well in *M. thermophila*

**Fig. 4:**
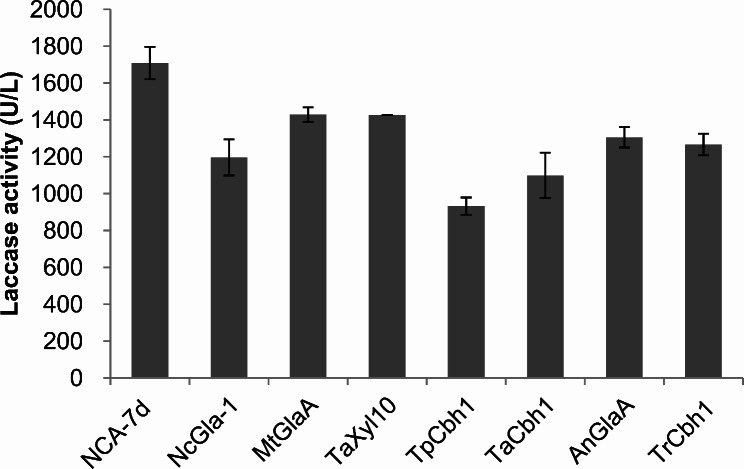
Influence of signal peptides on laccase production. The *M. thermophila* strains were cultured in fermentation medium at 45 °C and 200 rpm for 3 days

Protein secretion can also be affected by the presence of a signal peptide (SP). To further improve laccase production, we conducted an evaluation of several SPs selected from proteins with high secretion levels. These SPs included those from NcGla-1 [52, 53], MtGlaA [53], TaXyl10 and TaCbh1 [54], TpCbh1, AnGlaA [55], and TrCbh1 [56]. These SPs were tested for their ability to enhance laccase production in *M. thermophila*. The results showed that all of the SPs tested were effective in promoting laccase production, with activity levels comparable to or slightly lower than that of the native laccase SP (Fig. 4). While the other SPs did not show any significant improvement in laccase production, they were found to be functional in *M. thermophila*. These findings suggest that the use of these SPs could be a promising strategy for improving protein yields in biomanufacturing and highlight the versatility of *M. thermophila* in the field of biotechnology.

### High-level production of homologous and heterologous proteins

**Fig. 5:**
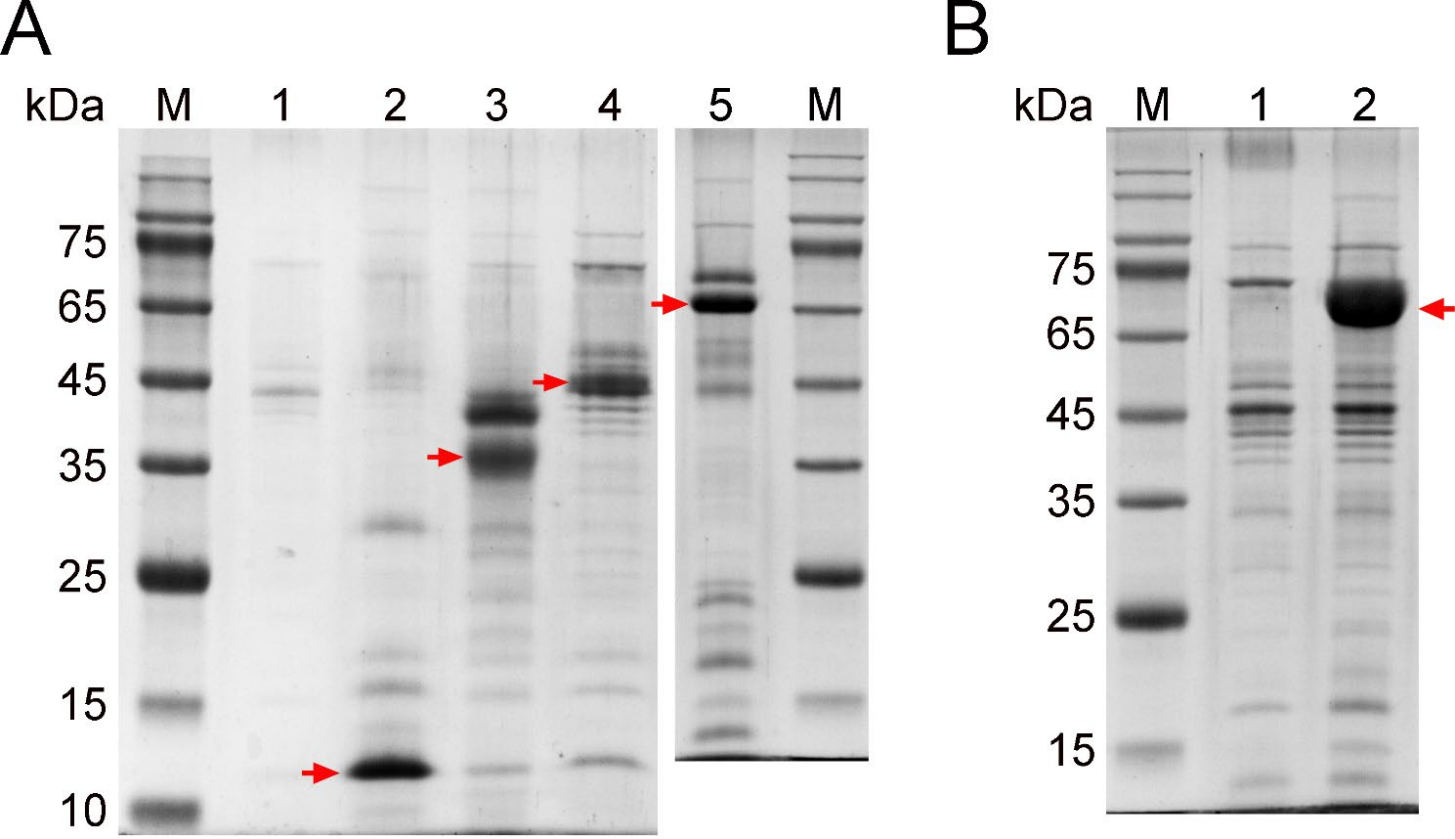
SDS-PAGE analysis of the extracellular proteins of the *M. thermophila* strains. (**A**) Overexpression of MtCbh-1, MtGh5-1, MtLPMO9B, and MtEpl1. Lane M, molecular mass standard; Lane 1, the parent ∆*Mtalp1* strain; Lane 2, *Mtepl1*-expressing strain; Lane 3, *MtLPMO9B*-expressing strain; Lane 4, *Mtgh5-1*-expressing strain; Lane 5, *Mtcbh-1*-expressing strain. Red arrows indicate the protein band corresponding to MtEpl1, MtLPMO9B, MtGh5-1, and MtCbh-1, respectively. (**B**) Overexpression of TrGlaA. Lane 1, the parent ∆*Mtalp1* strain; Lane 2, *TrglaA*-expressing strain. The red arrow indicates the protein band corresponding to TrGlaA. All strains were cultured in fermentation medium for 3 days

The enzymatic hydrolysis of plant biomass is a critical step in biorefinery processes, but it is hindered by the high cost of plant biomass-degrading enzymes (PBDEs). PBDEs, as environmentally friendly catalysts, can effectively convert plant residues into single sugars such as glucose. However, the current cost of PBDEs is prohibitive for industrial biorefinery applications. Using the laccase reporting system, we have demonstrated the effectiveness of the P*pdc* promoter, NCA-7d 5’UTR, and SP of *lcc1* (SP*lcc1*) in facilitating extracellular protein production. To evaluate the versatility of the system, we used it to overexpress several homologous proteins, including three PBDEs. The coding sequences of *Mtcbh-1*, *Mtgh5-1*, *MtLPMO9B*, and *Mtepl1* were cloned along with their respective terminators, excluding the SP-coding region. These sequences were then placed under the control of P*pdc*-NCA-7d-SP*lcc1* expression elements. The resulting constructs were then introduced into the ∆*Mtalp1* mutant, and the transformants were assayed for protein production. As shown in Fig. 5A, all four genes were effectively overexpressed, resulting in significant secretion of their respective proteins into the supernatant. Consistently, the cellobiohydrolase activity of the *Mtcbh-1*-overexpressing strain was significantly higher than that of the *∆Mtalp1* strain (6.09 ± 0.72 U/L vs. 1.39 ± 0.21 U/L, *P* < 0.001); and the endoglucanase activity of the *Mtgh5-1*-overexpressiong strain was significantly higher than that of the ∆*Mtalp1* strain (5.49 ± 0.26 U/mL vs. 1.50 ± 0.11 U/mL, *P* < 0.001). Furthermore, the LC-ESI-MS/MS analysis confirmed that the bands observed on the SDS-PAGE gel corresponded to MtCbh-1, MtGh5-1, MtLPMO9B, and MtEpl1 proteins, respectively (**Table S2**).

To test whether this system is effective in the production of heterologous proteins, we used it to express the glucoamylase gene *TrglaA* from *T. reesei*. The result showed that the TrGlaA was abundantly secreted into the supernatant (Fig. 5B) and exhibited significantly higher glucoamylase activity compared to the ∆*Mtalp1* strain (33.14 ± 1.37 U/mL vs. 2.02 ± 0.12 U/mL, *P* < 0.001). This finding highlights the adaptability of this system for the expression of heterologous proteins. In addition, the identity of the corresponding band on the SDS-PAGE gel as TrGlaA was confirmed through LC-ESI-MS/MS analysis (**Table S2**). This indicates that the system is a versatile protein production platform with the potential to produce a wide variety of proteins.

## Conclusions

A protein expression system utilizing the *Mtpdc* promoter, the NCA-7d 5’UTR, and the SP of *lcc1* has been successfully established in *M. thermophila*. This system enables the efficient expression of various proteins, including homologous proteins like MtCbh-1, MtGh5-1, MtLPMO9B, and MtEpl1, as well as a heterologous glucoamylase from *T. reesei*. The development of such a highly efficient protein expression system in *M. thermophila* holds great potential for diverse biotechnological applications. Further optimization and expansion of this system, together with rational fungal chassis design and engineering, are necessary to facilitate the production of a wide range of industrial enzymes and other proteins.

## Methods

### Strains, media, and growth conditions

*M. thermophila* ATCC 42464 was obtained from the American Type Culture Collection (ATCC). *Escherichia coli* Trans1-T1 (TransGen, Beijing, China) was used for plasmid construction and propagation, and was cultured in Luria-Bertani (LB) medium supplemented with 100 µg/mL ampicillin. *M. thermophila* strains were grown on Vogel’s minimal medium (MM) supplemented with 2% (w/v) sucrose at 37 °C for 7 days to obtain mature conidia. For flask culture, conidia from the *M. thermophila* strains were inoculated into 50 mL of fermentation medium [31] containing 4% (w/v) glucose to a final concentration of 1 × 10^6^ conidia/mL in a 100-mL Erlenmeyer flask. The culture was then incubated at 45 °C with continuous shaking at 200 rpm. Selection medium (1 × Vogel’s salts, 2 g/L sucrose, 182 g/L sorbitol, and 7.5 g/L agarose) was used to screen transformants after protoplast transformation.

### Construction of the *M. thermophila* ∆ *Mtalp1* strain

All primer sequences used in this study are listed in **Table ****S1**. Deletion of *Mtalp1* (MYCTH_2303011) using the CRISPR-Cas9 system was performed as previously described [18]. To construct the gRNA expression cassette for *Mtalp1*, the target DNA sequence in the plasmid U6p-*ArAceA*-sgRNA was replaced with that of *Mtalp1* (5’-GTCTACCGCGGCAAGTTCAG-3’) by PCR with paired primers alp1-gRNA-F/U6p-alp1-R, followed by self-recombination using a ClonExpress Ultra One Step Cloning Kit (Vazyme Biotech, Nanjing, China), resulting in plasmid U6p-*Mtalp1*-sgRNA. The gRNA expression cassette was amplified with U6p-F/gRNA-R. To construct the donor DNA sequences, the 5’ and 3’ flanking fragments of *Mtalp1* were amplified with paired primers alp1-up-F/R and alp1-down-F/R, respectively, using *M. thermophila* genomic DNA as the template. The selectable marker cassette P*trpC*-*hph* was amplified with paired primers PtrpC-F/hph-R. The 5’ and 3’ fragments and P*trpC*-*hph* were assembled and inserted into the pEASY-Blunt Simple cloning vector (TransGen Biotech, Beijing, China) to generate donor-*Mtalp1*. The 5′-P*trpC*-*hph*-3′ cassette was amplified using the primers alp1-up-F/alp1-down-R. The Cas9-expression PCR cassette P*tef1*-*Cas9*-T*trpC* was amplified from the plasmid p0380-*bar*-P*tef1*-*Cas9*-T*trpC* [18] using Ptef-cas-F/TtrpC-cas-R. All constructed plasmids were verified by sequencing. For the subsequent target gene deletion, a total of 10 µg PCR products of the Cas9-expression cassette P*tef1*-*Cas9*-T*trpC*, the gRNA expression cassette U6p-*Mtalp1*-sgRNA, and the corresponding donor fragment were mixed at the same molar concentration ratio and cotransformed into protoplasts of the wild-type strain of *M. thermophila*.

### Transformation of *M. thermophila* protoplasts

Conidia were harvested and spread onto MM plates covered with cellophane. After incubation at 37 °C for 12 h, mycelia were collected and used for the preparation of protoplasts. Protoplasts of *M. thermophila* were prepared as previously described [57]. Transformants were screened using selection medium supplemented with 100 µg/mL hygromycin B for the ∆*Mtalp1* mutant or 100 µg/mL Geneticin for target gene expression, and incubated at 37 °C for 4 days. The confirmation of transformants was performed by PCR analysis.

### Overexpression of the laccase gene in *M. thermophila*

To overexpress *lcc1* (MYCTH_51627) under the control of the strong constitutive promoters of *M. thermophila*, the coding region of *lcc1* and its terminator (*lcc1*-T*lcc1*) were amplified by PCR using paired primers lcc-F/lcc-T-R. The promoters of *Mttef1* (MYCTH_2298136), *Mthsp30* (MYCTH_87219) and *Mtpdc* (MYCTH_112121) were amplified with paired primers Ptef-F/R, Phsp-F/R, and Ppdc-F/R, respectively. To obtain the promoter of *Mtgpd* (MYCTH_2311855), two fragments were amplified using primers Pgpd-F1/R1 and Pgpd-F2/R2, respectively, and then fused by PCR to remove the introns present in the 5’UTR of *Mtgpd* promoter. The P*trpC*-*neo* cassette was amplified using the primers PtrpC-F/neo-T-R. The P*trpC*-*neo* cassette, *lcc1*-T*lcc1* cassette, and the respective promoter were assembled into the pEASY-Blunt Simple cloning vector using the ClonExpress Ultra One Step Cloning Kit, resulting in plasmids pPtef1-lcc1, pPhsp-lcc1, pPpdc-lcc1 and pPgpd-lcc1, respectively. The P*trpC*-*neo*-P*tef1*/P*hsp*/P*pdc*/P*gpd*-*lcc1*-T*lcc1* cassettes were amplified with primers PtrpC-F/lcc-T-R and used for protoplast transformation of the ∆*Mtalp1* mutant.

To overexpress *lcc1* under the control of the P*pdc* promoter and different 5’UTRs, the 5’UTR sequences of *Mtcbh-1* (MYCTH_109566) and *Thermoascus aurantiacus LPMO9N* (*TaLPMO9N*, JGI: Theau2|Transcript ID: 668012) were amplified from their respective genomes using paired primers Mtcbh1-5’-F/R and 9 N-5’-F/R. These fragments were then assembled with the fragment amplified from plasmid pPpdc-lcc1 using primers Mtpdc-R/lcc-F to replace the 5’UTR of *Mtpdc* in pPpdc-lcc1. To replace the 5’UTR of *Mtpdc* in plasmid pPpdc-lcc1 with NCA-7d and that of *T. reesei cbh1* (*Trcbh1*, TRIREDRAFT_123989), we amplified fragments from plasmid pPpdc-lcc1 using paired primers Trcbh-5’-F/R and NCA-7d-F/R, respectively. Self-recombination was then performed to generate plasmids pNCA-7d-lcc1 and pTc-5’-lcc1, respectively. The P*trpC*-*neo*-P*pdc*-5’UTRs-*lcc1*-T*lcc1* cassettes were amplified with primers PtrpC-F/lcc-T-R and used for protoplast transformation of the ∆*Mtalp1* mutant.

To replace the signal peptide (SP)-encoding sequence in plasmid pNCA-7d-lcc1, we amplified the fragments from pNCA-7d-lcc1 using paired primers NcGla-1-SP-F/R, MtGlaA-SP-F/R, TaXyl10-SP-F/R, TaCbh1-SP-F/R, TpCbh1-SP-F/R, AnGlaA-SP-F/R, TrCbh1-SP-F/R, respectively. After self-recombination, the SP-encoding sequence of *lcc1* were replaced with that of *Neurospora crassa* glucoamylase NcGla-1 (NCU01517), *M. thermophila* glucoamylase MtGlaA (MYCTH_72393), *T. aurantiacus* xylanase TaXyl10 (JGI: Theau2|protein ID: 636296) and cellobiohydrolase TaCbh1 (JGI: Theau2|protein ID: 674763), *Talaromyces piceae* cellobiohydrolase TpCbh1 (ATQ35967.1), *A. niger* glucoamylase AnGlaA (An03g06550), and *T. reesei* cellobiohydrolase TrCbh1, respectively. The P*trpC*-*neo*-P*pdc*-NCA-7d-SPs-*lcc1*-T*lcc1* cassettes were amplified with primers PtrpC-F/lcc-T-R and used for protoplast transformation of the ∆*Mtalp1* mutant.

### Overexpression of homologous genes in *M. thermophila*

The coding regions of *Mtcbh-1* (cellobiohydrolase), *Mtgh5-1* (MYCTH_86753, endoglucanase), *MtLPMO9B* (MYCTH_80312, lytic polysaccharide monooxygenase), and *Mtepl1* (MYCTH_2090891, eliciting plant response-like protein), along with their respective terminators, were amplified with paired primers Mtcbh1-F/Mtcbh1-T-R, Mtgh5-F/ Mtgh5-T-R, Mt9B-F/Mt9B-T-R, and Mtepl-F/Mtepl-T-R. These fragments were then assembled with the fragment amplified from plasmid pNCA-7d-lcc1 using primers lcc-SP-R/Vec-F. The P*trpC*-*neo*-P*pdc*-NCA-7d-SP*lcc1*-*Mtcbh-1/Mtgh5-1/MtLPMO9B/Mtepl1*-Ter cassettes were amplified with primers PtrpC-F/Ter-R and used for protoplast transformation of the ∆*Mtalp1* mutant.

To identify the proteins, the protein band was excised from the gel, digested with trypsin, and sequenced using liquid chromatography/electrospray ionization tandem mass spectrometry (LC-ESI-MS/MS) at the Institute of Apicultural Research, Chinese Academy of Agricultural Sciences.

### Overexpression of *TrglaA* gene in *M. thermophila*

The coding region of *TrglaA* (TRIREDRAFT_1885) was amplified from the *T. reesei* genome using paired primers TrGlaA-F/TrGlaA-R and then assembled with the fragment amplified from plasmid pNCA-7d-lcc1 using primers lcc-T-F/lcc-SP-R. The P*trpC*-*neo*-P*pdc*-NCA-7d-SP*lcc1*-TrGlaA-T*lcc1* cassettes were amplified with primers PtrpC-F/Ter-R and used for protoplast transformation of the ∆*Mtalp1* mutant. LC-ESI-MS/MS was used for the identification of TrGlaA.

### Enzyme activity determination

Laccase activity was determined by the methods of oxidation of 2,2’-azino-bis(3-ethylbenzothiazoline-6-sulfonic acid) (ABTS) as described previously [45] with some modification. The assay system was composed of 20 µL supernatant and 180 µL 100 mM sodium acetate buffer (pH 5.0) containing 1 mM ABTS at room temperature. Oxidation of ABTS was monitored by determining the increase of absorbance at 420 nm. One unit (U) of enzyme activity was defined as the amount of the laccase that oxidized 1 µmol of ABTS per minute.

The *β*-glucanase activity was determined according to the 3,5-dinitrosalicylic acid (DNS) method as previously described [58], using 1% carboxymethylcellulose sodium (CMC-Na) as substrate. The glucoamylase activity was determined using soluble starch as the substrate. Reactions containing 900 µL of 1% soluble starch in Na_2_HPO_4_-citric acid (pH 5.5) and 100 µL of appropriately diluted enzyme solution were incubated at 60 °C for 10 min, using the DNS method to detect the amount of reducing sugar in the reaction. The activity of pNPCase was measured by adding 50 µL of appropriately diluted enzyme solution to 150 µL of 1 mM *p*-nitrophenyl-*β*-D-cellobioside (pNPC) in 100 mM sodium acetate buffer (pH 5.5). The mixture was incubated at 50 °C for 30 min. Then 100 µL of 1.0 M Na_2_CO_3_ was added to stop the reaction, and the absorbance was measured at 420 nm. One unit (U) of enzyme activity was defined as the amount of enzyme that released 1 µmol of glucose per minute.

### Electronic supplementary material

Below is the link to the electronic supplementary material.

Supplementary Material 1

## Data Availability

All of the data generated in this study are available and have been already included in the main text and supplemental material.
