# Supplementary material for: Development of an efficient protein expression system in the thermophilic fungus Myceliophthora thermophila
Source: Microb Cell Fact. 2023 Nov 16;22:236. doi: 10.1186/s12934-023-02245-5 (PMC10652509; doi:10.1186/s12934-023-02245-5)
Supplement: Supplementary file 1 — Supplementary Material 1 [file 12934_2023_2245_MOESM1_ESM.docx]

**Table S1. Primers used in this study**

| Primers | Sequences (5'–3') |
| --- | --- |
| alp1-gRNA-F | CCTCGTCTACCGCGGCAAGTTCAGGTTTTAGAGCTAGAAATAGCAAG |
| U6p-alp1-R | AACCTGAACTTGCCGCGGTAGACGAGGAAAGAAAGAAAAGAAGAGG |
| alp1-up-F | CTAGTCCTCGCAGCAAACTCG |
| alp1-up-R | TTATAGGTTAATGTCATGATCTGAACTTGCCGCGGTAGAC |
| alp1-down-F | GTAAAGATAATGCTAAATCACACCACCTACACCTACGACAC |
| alp1-down-R | GTTGGCGGCGTTCTGGTTCTCG |
| U6p-F | AGGATCGGTGGAGTGAAG |
| gRNA-R | AAAAGCACCGACTCGGTGCCAC |
| PtrpC-F | ATCATGACATTAACCTATAAAAATAGG |
| hph-R | TGATTTAGCATTATCTTTAC |
| Ptef-cas-F | TCCGAGGTTCGACATCAG |
| TtrpC-cas-R | GCTCCCTCTAAACAAGTG |
| lcc-F | ATGAAGTCCTTCATCAGCGC |
| lcc-T-R | CCTCTAAACAAGTGTACCTGGCAGTCCTTGCAGATACTGC |
| Ptef-F | CATGCATGTTGCATGATGATGTAATGGCAGCACCTTGACG |
| Ptef-R | GCGCTGATGAAGGACTTCATTTTGACGGTATTTGTGTTCTGAAGAACGAAACTGGC |
| Phsp-F | CATGCATGTTGCATGATGATAAGGACTGGCCGATGCAGTC |
| Phsp-R | GCGCTGATGAAGGACTTCATGTTGCGTTGCTGTTGTTTTTTAAGCTGTTGCCGAGTTGCTG |
| Ppdc-F | CATGCATGTTGCATGATGATCGAGTGTACTCCGTAAGGAG |
| Ppdc-R | GCGCTGATGAAGGACTTCATGTCTGTTGTTGGCGATGTTTG |
| Pgpd-F1 | CATGCATGTTGCATGATGATGATGGAGGACTACCATCGTC |
| Pgpd-R1 | CAAGCATTGCCCAGTGTATGC |
| Pgpd-F2 | GCATACACTGGGCAATGCTTGGCCGTCCGCGCTCTGATAACCAC |
| Pgpd-R2 | GCGCTGATGAAGGACTTCATTTTGATTTCTGGAGGGATGAGGGAGCTTTG |
| neo-T-R | ATCATCATGCAACATGCATG |
| Mtpdc-R | TACAAAGACTTGAAAACGGCCTC |
| Mtcbh1-5’-F | GCCGTTTTCAAGTCTTTGTAGGCTGGCCGGCTGACCTTTGTGAC |
| Mtcbh1-5’-R | GCGCTGATGAAGGACTTCATGGCGGAAGAGGATCAGCACACAG |
| 9N-5’-F | GCCGTTTTCAAGTCTTTGTACAATGTTGGAATATAAATATGGCTTGTCTAACTCAACC |
| 9N-5’-R | GCGCTGATGAAGGACTTCATCTTTCAAATAGGATGTGCCAATTCGATTGCGATGG |
| Trcbh-5’-F | ACACCATCTTTTGAGGCACAGAAACCCAATAGTCAACCGCGGACTGCGCATCATGAAGTCCTTCATCAGCGC |
| Trcbh-5’-R | TGTGCCTCAAAAGATGGTGTACAAGTCTCCTGGAGGATCTGAGTTGTACAAAGACTTGAAAACGGC |
| NCA-7d-F | CAATCAATCATCAACACATCATCAAGACACCACCATGAAGTCCTTCATCAGCGC |
| NCA-7d-R | GATGTGTTGATGATTGATTGTTGATGTTGTGATGATGATTGATTTTGATTTTTGCCTACAAAGACTTGAAAACGGC |
| NcGla-1-SP-F | TCTCTTCGCTCCTCGTCGTGGGCGCCGCCTTCCAGGCCGTGCTCGGTGCCCCTCCATCCACCCCTGAG |
| NcGla-1-SP-R | CACGACGAGGAGCGAAGAGACGAGATGCATGGTGGTGTCTTGATGATGTGTTG |
| MtGlaA-SP-F | GTCCAGACGGTCTTGGGCCGTCCGGCCACCCTCTCAAAGCGGGCCCCTCCATCCACCCCTGAG |
| MtGlaA-SP-R | GGCCCAAGACCGTCTGGACGGCCCAGGCGCCGAGGACAGCGAGCGACGAGAGAGCGTGCATGGTGGTGTCTTGATGATGTG |
| AnGlaA-SP-F | CCTCGTCTGCACAGGGTTGGCAAATGTGATTTCCAAGCGCGCCCCTCCATCCACCCCTGAG |
| AnGlaA-SP-R | CAACCCTGTGCAGACGAGGCCGCTCAGGGCGAGTAGAGATCGGAACGACATGGTGGTGTCTTGATGATGTGTTG |
| TaCbh1-SP-F | ATGTATCAGCGCGCTCTTCTCTTCTCTTTCTTCCTCGCCGCCGCCCGCGCGGCCCCTCCATCCACCCCTGAG |
| TaCbh1-SP-R | GAGAAGAGCGCGCTGATACATGGTGGTGTCTTGATGATGTGTTG |
| TaXyl10-SP-F | ACTTCACTCCTGCTAGCTCCCTTCGCAGCTGCGAGCCCTATCCTCGAGGAACGCGCCCCTCCATCCACCCCTGAG |
| TaXyl10-SP-R | GGAGCTAGCAGGAGTGAAGTAAGTAGGATCGTTGGTCTAACCATGGTGGTGTCTTGATGATGTGTTG |
| TpCbh1-SP-F | CTCCACTTCCCTTTACTTCGCTGTCCAAGCAGCCCCTCCATCCACCCCTGAG |
| TpCbh1-SP-R | CGAAGTAAAGGGAAGTGGAGAAAAGAAGAGCGCGCTGGAACATGGTGGTGTCTTGATGATGTGTTG |
| TrCbh1-SP-F | GTCATCTCGGCCTTCTTGGCCACAGCTCGTGCTGCCCCTCCATCCACCCCTGAG |
| TrCbh1-SP-R | GCCAAGAAGGCCGAGATGACGGCCAACTTCCGATACATGGTGGTGTCTTGATGATGTGTTG |
| lcc-SP-R | AGCAGCAACGCTAGGGGTGAG |
| Vec-F | CAGGTACACTTGTTTAGAGG |
| Mtepl-F | TCACCCCTAGCGTTGCTGCTACCACAGGTTCGTCACCTCC |
| Mtepl-T-R | CCTCTAAACAAGTGTACCTGGAAGAGGTGAGCGTGGAGGC |
| Mtcbh1-F | TCACCCCTAGCGTTGCTGCTCAGAACGCCTGCACTCTGAC |
| Mtcbh1-T-R | CCTCTAAACAAGTGTACCTGGACTTTGATCAACTACGATGCG |
| Mtgh5-F | TCACCCCTAGCGTTGCTGCTCAAAGTGGTCCGTGGCAGCAATG |
| Mtgh5-T-R | CCTCTAAACAAGTGTACCTGTCCTCAGGAACGCACACTGC |
| Mt9B-F | TCACCCCTAGCGTTGCTGCTCACGCGACCTTCCAGGCCCTC |
| Mt9B-T-R | CCTCTAAACAAGTGTACCTGAAAGAGCTTGCGGACAAGGG |
| Ter-R | CCTCTAAACAAGTGTACCTG |
| TrGlaA-F | TCACCCCTAGCGTTGCTGCTAGACCAGGATCAAGCGGTCTG |
| TrGlaA-R | CTCCCTCGCTCAGTGGTGGTGGTGGTGGTGCGACTGCCAGGTGTCCTCCTTG |

**Table S2. Unique sequence identification by LC-ESI-MS/MS**

| **Protein** | **Peptide** | **-10lgP** | **Mass** | **ppm** | **m/z** | **RT** | **Fraction** | **Scan** | **Intensity** | **#Spec** | **PTM** |
| --- | --- | --- | --- | --- | --- | --- | --- | --- | --- | --- | --- |
| MtGh5-1 | K.YAVLDPHNYGR.Y | 60.08 | 1303.63 | 1.3 | 652.82 | 14.31 | 2 | 3621 | 7.94E+08 | 24 |  |
|  | R.YYGNIITDTNAFR.T | 55.03 | 1546.74 | 0.1 | 774.37 | 19.46 | 2 | 5407 | 1.39E+07 | 2 |  |
|  | R.VVGATQWLR.A | 44.84 | 1028.57 | 1 | 515.29 | 16.74 | 2 | 4563 | 5.33E+08 | 10 |  |
|  | R.IDFSMER.L | 35.54 | 896.40 | 0 | 449.21 | 16.05 | 2 | 4321 | 2.91E+08 | 5 |  |
|  | R.TFWTNLAK.Q | 32.6 | 979.51 | 0.2 | 490.76 | 17.9 | 2 | 4935 | 3.81E+06 | 6 |  |
|  | R.IDFSM(+15.99)ER.L | 23.46 | 912.40 | 0.6 | 457.20 | 13.22 | 2 | 3170 | 3.08E+08 | 11 | Oxidation (M) |
| MtEpl1 | K.YGWQTQGQVK.G | 48.35 | 1193.58 | 0.9 | 597.79 | 16.76 | 1 | 4985 | 5.81E+09 | 19 |  |
| MtCbh-1 | K.AAFGDVTDFQDKGGM(+15.99)VQM(+15.99)GK.A | 80.08 | 2132.95 | 1.1 | 711.99 | 15.01 | 1 | 4652 | 2.76E+07 | 5 | Oxidation (M) |
|  | K.FINGEANVENWQSSTNDANAGTGK.Y | 77.88 | 2523.12 | 0.8 | 842.05 | 16.28 | 1 | 5259 | 2.08E+07 | 3 |  |
|  | K.NSAGELSEIKR.F | 68.87 | 1202.62 | 0.7 | 602.32 | 13.46 | 1 | 3863 | 1.82E+09 | 22 |  |
|  | K.KITVVTQFLK.N | 62.08 | 1175.72 | 0.4 | 588.87 | 17.05 | 1 | 5628 | 1.87E+08 | 14 |  |
|  | K.AAFGDVTDFQDK.G | 61.43 | 1312.59 | 0.5 | 657.30 | 18.31 | 1 | 6172 | 1.64E+09 | 11 |  |
|  | K.GMTVDTTKK.I | 57.93 | 979.50 | 0.2 | 490.75 | 10.92 | 1 | 2688 | 1.80E+08 | 5 |  |
|  | K.NSAGELSEIK.R | 56.46 | 1046.52 | 0.7 | 524.26 | 13.52 | 1 | 3898 | 2.78E+08 | 6 |  |
|  | R.TYLMESDTK.Y | 54.58 | 1086.49 | 0.7 | 544.25 | 13.67 | 1 | 3981 | 3.70E+08 | 13 |  |
|  | K.GQYSTNIGSR.T | 51.98 | 1081.51 | 0.7 | 541.76 | 12.56 | 1 | 3356 | 9.24E+08 | 17 |  |
|  | R.TYLM(+15.99)ESDTK.Y | 49.96 | 1102.48 | 0.2 | 552.25 | 12.36 | 1 | 3261 | 8.59E+08 | 11 | Oxidation (M) |
|  | K.GM(+15.99)TVDTTKK.I | 45.97 | 995.49 | 0.4 | 498.75 | 3.24 | 1 | 714 | 1.15E+08 | 29 | Oxidation (M) |
|  | K.GM(+15.99)TVDTTK.K | 43.6 | 867.40 | -0.1 | 434.70 | 10.78 | 1 | 2636 | 1.59E+07 | 2 | Oxidation (M) |
|  | K.GMTVDTTK.K | 42.87 | 851.40 | 0 | 426.71 | 10.68 | 1 | 2586 | 1.11E+08 | 4 |  |
|  | K.ITVVTQFLK.N | 39.75 | 1047.63 | 1 | 524.82 | 19.65 | 1 | 6666 | 4.18E+08 | 4 |  |
|  | K.AAFGDVTDFQDKGGM(+15.99)VQMGK.A | 39.65 | 2116.95 | 0.7 | 706.65 | 16.58 | 1 | 5401 | 3.61E+06 | 1 | Oxidation (M) |
|  | K.GGMVQMGK.A | 37.9 | 806.379 | -0.1 | 404.19 | 12.34 | 1 | 3246 | 5.28E+07 | 3 |  |
|  | K.FVTKGQYSTNIGSR.T | 34.89 | 1556.79 | 2.7 | 779.40 | 12.88 | 1 | 3537 | 5.80E+06 | 2 |  |
|  | K.GGM(+15.99)VQMGK.A | 33.67 | 822.37 | -0.3 | 412.19 | 10.13 | 1 | 2373 | 7.02E+07 | 6 | Oxidation (M) |
|  | R.FYVQNGK.V | 31.9 | 854.42 | 0.1 | 428.22 | 12.2 | 1 | 3182 | 1.46E+08 | 2 |  |
|  | K.GGMVQM(+15.99)GK.A | 28.37 | 822.37 | -0.1 | 412.19 | 12.37 | 1 | 3264 | 6.37E+06 | 2 | Oxidation (M) |
|  | R.QKAAFGDVTDFQDK.G | 26.86 | 1568.74 | 1.3 | 785.38 | 14.52 | 1 | 4423 | 1.27E+06 | 1 |  |
|  | K.TFYGKGM(+15.99)TVDTTK.K | 13.97 | 1463.69 | 0.7 | 732.85 | 12.29 | 1 | 3220 | 1.57E+06 | 1 | Oxidation (M) |
| TrGlaA | R.FWVSSGGYVDSNINTNEGR.T | 86.54 | 2100.95 | 1.3 | 1051.48 | 18.7 | 1 | 6984 | 5.40E+08 | 8 |  |
|  | R.SIYGVNKGIPAGAAVAIGR.Y | 66.71 | 1813.02 | 0.3 | 605.34 | 16.79 | 1 | 5829 | 9.29E+06 | 4 |  |
|  | K.YVPADGSLAEQFDR.N | 62.34 | 1566.73 | 1.1 | 784.37 | 18.92 | 1 | 7131 | 5.45E+09 | 11 |  |
|  | K.FELTLKPFTGNWGRPQR.D | 59.17 | 2046.07 | 1.1 | 683.03 | 19.5 | 1 | 7500 | 1.70E+08 | 6 |  |
|  | R.FTETYDAGLQR.R | 50.06 | 1299.60 | 0.2 | 650.81 | 14 | 1 | 4222 | 6.13E+09 | 17 |  |
|  | K.GIPAGAAVAIGR.Y | 43.42 | 1051.61 | 1.3 | 526.81 | 16.05 | 1 | 5408 | 4.92E+09 | 16 |  |
|  | R.AIALIGYSK.W | 33.78 | 934.54 | 0.9 | 468.28 | 16.88 | 1 | 5875 | 1.56E+09 | 10 |  |
|  | R.DSALVFK.N | 32.06 | 778.42 | 0 | 390.21 | 15.75 | 1 | 5246 | 1.49E+09 | 3 |  |
|  | R.SIYGVNK.G | 28.3 | 779.41 | 0.2 | 390.71 | 12.72 | 1 | 3511 | 1.32E+09 | 3 |  |
|  | K.VVVDSFR.S | 27.68 | 820.44 | -0.3 | 411.23 | 16 | 1 | 5373 | 1.38E+09 | 13 |  |
| MtLPMO9B | K.ATDPGILVNIHAPLSGYTVPGPAVYSGGSTK.K | 106.9 | 3038.57 | -1.4 | 1520.29 | 18.28 | 1 | 9557 | 3.23E+10 | 22 |  |
|  | R.LPASNSPVTDVTSNAIR.C | 88.65 | 1740.90 | 2.1 | 871.45 | 13.34 | 1 | 6155 | 3.59E+10 | 27 |  |
|  | K.NPSGGSGDDDYWGTK.D | 87.34 | 1554.62 | 1.1 | 778.31 | 12.33 | 1 | 5348 | 2.43E+09 | 3 |  |
|  | K.AGSTVTVEMHQQPGDR.S | 84.32 | 1711.79 | 3.6 | 856.90 | 11.01 | 1 | 4365 | 4.03E+09 | 10 |  |
|  | K.ATDPGILVNIHAPLSGYTVPGPAVYSGGSTKK.A | 83.05 | 3166.66 | -0.2 | 1056.56 | 16.45 | 1 | 8338 | 6.09E+08 | 3 |  |
|  | K.AGSTVTVEM(+15.99)HQQPGDR.S | 74.85 | 1727.78 | 1.9 | 864.90 | 10.98 | 1 | 4336 | 1.32E+10 | 35 | Oxidation (M) |
|  | K.VSDAASADGSSGWFK.V | 71.43 | 1483.65 | 1.9 | 742.83 | 13.29 | 1 | 6116 | 1.08E+10 | 7 |  |
|  | K.M(+15.99)NVKIPADLPSGDYLLR.A | 68.47 | 1917.00 | 1.5 | 640.00 | 15.38 | 1 | 7601 | 1.58E+08 | 3 | Oxidation (M) |
|  | K.IPADLPSGDYLLR.A | 65.37 | 1428.76 | 0.4 | 477.26 | 16.3 | 1 | 8232 | 4.47E+10 | 18 |  |
|  | K.VFEDGWAK.N | 43.92 | 950.44 | -0.3 | 476.23 | 13.61 | 1 | 6360 | 7.39E+09 | 7 |  |
|  | K.MNVKIPADLPSGDYLLR.A | 41.35 | 1901.00 | 0.9 | 634.67 | 16.71 | 1 | 8499 | 5.46E+06 | 1 |  |
